# Plasma Cytokines Profile in Subjects with Alzheimer’s Disease: Interleukin 1 Alpha as a Candidate for Target Therapy

**DOI:** 10.31661/gmj.v10i0.1974

**Published:** 2021-01-25

**Authors:** Meisam Mahdavi, Saeed Karima, Shima Rajaei, Vajihe Aghamolaii, Hossein Ghahremani, Reza Ataei, Hessam Sepasi Tehrani, Somayeh Mahmoodi Baram, Abbas Tafakhori, Behnam Safarpour Lima, Somayeh Shateri, Hamid Fatemi, Farzad Mokhtari, Abdolrahim Nikzameer, Amir Yarhosseini, Ali Gorji

**Affiliations:** ^1^Department of Clinical Biochemistry, School of Medicine, Shahid Beheshti University of Medical Sciences (SBMU), Tehran, Iran; ^2^Department of Clinical Research, HealthWeX Co., Ltd., ON, Canada; ^3^Department of Psychiatry, School of Medicine, Roozbeh Hospital, Tehran University of Medical Sciences, Tehran, Iran; ^4^Department of Neurology, School of Medicine, Iranian Center of Neurological Research, Neuroscience Institute, Imam Khomeini Hospital, Tehran University of Medical Sciences, Tehran, Iran; ^5^Department of Neurology, School of Medicine, Imam Hossein Hospital, Shahid Beheshti University of Medical Science, Tehran, Iran; ^6^Department of Neurology and Department of Neurosurgery, Westfälische Wilhelms-Universität Münster, Münster, Germany

**Keywords:** Alzheimer’s Disease, Aβ Peptide, Pro-/Anti-inflammatory Cytokines, IL-1α, NPI-Q

## Abstract

**Background:**

Alzheimer’s disease (AD) is the main cause of the neurodegenerative disorder, which is not detected unless the cognitive deficits are manifested. An early prediagnostic specific biomarker preferably detectable in plasma and hence non-invasive is highly sought-after. Various hypotheses refer to AD, with amyloid-beta (Aβ) being the most studied hypothesis and inflammation being the most recent theory wherein pro-and anti-inflammatory cytokines are the main culprits.

**Materials and Methods:**

In this study, the cognitive performance of AD patients (n=39) was assessed using mini-mental state examination (MMSE), AD assessment scale-cognitive subscale (ADAS-cog), and clinical dementia rating (CDR). Their neuropsychiatric symptoms were evaluated through neuropsychiatric inventory–questionnaire (NPI-Q). Moreover, plasma levels of routine biochemical markers, pro-/anti-inflammatory cytokines such as tumor necrosis factor α (TNF-α), interleukin-1 α (IL-1α), IL-1β, IL-2, IL-4, IL-6, IL-8, IL-12p70, IL-10, Interferon-gamma, chemokines, including prostaglandin E2 (PGE-2), monocyte chemoattractant protein-1, interferon gamma-induced protein 10, Aβ peptide species (42, 40) and Transthyretin (TTR) were measured.

**Results:**

Our results revealed that Aβ 42/40 ratio and TTR were correlated (r=0.367, P=0.037). IL-1α was directly correlated with ADAS-cog (r=0.386, P=0.017) and Aβ 40 (r=0.379, P=0.019), but was inversely correlated with IL-4 (r=-0.406, P=0.011). Negative correlations were found between MMSE and PGE2 (r=-0.405, P=0.012) and TNF-α/ IL-10 ratio (r=-0.35, P=0.037). CDR was positively correlated with both PGE2 (r=0.358, P=0.027) and TNF-α (r=0.416, P=0.013). There was a positive correlation between NPI-caregiver distress with CDR (r=0.363, P=0.045) and ADAS-cog (r=0.449, P=0.019).

**Conclusion:**

Based on the observed correlation between IL-1α, as a clinical moiety, and ADAS-cog, as a clinical manifestation of AD, anti-IL-1α therapy in AD could be suggested.

## Introduction

Alzheimer’s disease (AD) is histopathologically recognized by the aggregation of extracellular amyloid-beta (Aβ) plaques and intracellular neurofibrillary tangles (NFTs). Despite unknown etiology, different hypotheses have been attributed to AD pathologies such as the Aβ peptide cascade and neuroinflammation involvement [1,2,3]. As per the Aβ peptide cascade hypothesis, abnormal accumulation of senile plaques in the brain, which stems from an imbalance between Aβ peptide production and clearance, accounts for synaptic loss and neuronal death [4]. Recently, it has been revealed that Aβ peptide accumulation is mainly due to impaired clearance rather than increased production of amyloid assemblies [5]. Aβ peptide deposition in the brain triggers glial activation and the release of pro-inflammatory cytokines and chemokines [6]. Brain exposure to such molecules culminates into synaptic dysfunction and eventually expedites the neurodegenerative processes in AD [7]. Many studies have addressed the diagnostic and therapeutic aspects of inflammatory molecules in AD [8]. However, finding a suitable and prospective candidate for AD diagnosis and treatment has been challenging. Plasma level of tumor necrosis factor-α (TNF-α) has a crucial role in triggering neuroinflammation through glial activation, is maintained at a low level in cognitively normal individuals, while it increases in patients with AD and mild cognitive impairment (MCI) [9]. In vivo studies on mice have demonstrated that TNF-α and Interferon-gamma (INF-γ) increase Aβ peptide production and lead to a decline in the microglial clearance of Aβ aggregates [10]. Moreover, a substantial rise in the plasma level of INF-γ, as an immune-regulatory cytokine, has been observed in AD patients, particularly in those who are at mild or severe stages [11]. Interleukin-(IL-)1α and IL-1β are strong pro-inflammatory cytokines involved in neuroinflammation as well as a learning process and memory [12]. While in patients with AD, the circulating level of IL-1 α remains controversial, overexpression of IL-1β has been observed [13,14]. IL-1β stimulates IL-6 release, astrocyte proliferation, and neuronal growth factor synthesis [15]. Clinical studies have shown that in patients with AD, the IL-6 plasma level is four times as high as that of normal healthy individuals [16]. The serum level of IL-12p70, a pro-inflammatory cytokine produced by dendritic cells and macrophages, rises in AD patients [17,18]. It has also been claimed that IL-2 is engaged in AD pathology since its level notably increases in AD. IL-10, as a multi-functional anti-inflammatory cytokine, is considered to be strongly involved in AD to the point where it is negatively correlated with the cerebrospinal fluid (CSF) level of Aβ peptide content [19]. Prostaglandins (PGs) are involved in memory deficit, and it has been found that the CSF level of PGE2 in patients with early AD is significantly higher than normal controls [20]. During inflammation, interferon gamma-induced protein 10 (IP-10) is also produced in response to INF-γ activity [21]. Monocyte chemoattractant protein-1 (MCP-1), a highly potent chemokine, regulates monocyte/ macrophages migration and infiltration. It has been illustrated that its CSF and plasma levels increase in AD [22,23]. Moreover, since microglia are engaged in the clearance of Aβ aggregates, it has been proposed that MCP-1-related inflammation is associated with Aβ peptide burden [24]. There are inconsistent results in the literature regarding the levels and roles of inflammatory mediators in AD, possibly due to the diverse and pleiotropic effects of cytokines and chemokines [25]. The present study aimed to provide a clear and comprehensive picture of possible correlations in AD patients. To meet that end, we investigated the correlation between plasma levels of pro- and anti-inflammatory cytokines, chemokines, Transthyretin (TTR), and amyloid species with cognitive functions and neuropsychiatric symptoms assessed through mini-mental state examination (MMSE), AD assessment scale-cognitive subscale (ADAS-cog), clinical dementia rating (CDR), and neuropsychiatric inventory–questionnaire (NPI-Q).

## Materials and Methods

### Participants and Eligibility Criteria

AD patients clinically diagnosed by NINCDS/ ADRDA criteria were recruited. Of the 93 subjects enrolled between October 2017 and April 2019, 39 individuals satisfied the eligibility criteria and entered the study. Subjects aged 65-85 years range with body mass index between 18 and 25 kg/m2, MMSE ranging from 10 to 25, a positive DSM-IV test, and Beck test score less than or equal to 12 were included. Subsequently, patients with a history of peptic ulcer, gastrointestinal surgery or bleeding, immunogenic viral or bacterial infections, chronic cardiovascular diseases, liver and kidney failures, Parkinson’s disease, other types of dementia, alcohol, and substance abuse were excluded. Those who took salicylate, coumarin derivatives, acetylcholinesterase and cholinergic antagonists, diuretics, estrogen, deprenyl, vitamin E, antipsychotics, NSAIDs, and systemic corticosteroids were also excluded. Other cognitive tests, including CDR and ADAS-cog, were applied to all participants. Moreover, neuropsychiatric symptoms were evaluated by the NPI-Q on both severity and caregiver distress scales.

### Blood Sampling

Fasting blood samples were collected (7-11 a.m.) into plain (serum) and EDTA/Citrate- containing tubes. To confirm exclusion criteria, before the study, the following blood tests were done on the patients’ sera: complete blood count (CBC), erythrocyte sedimentation rate (ESR), prothrombin time (PT), activated partial thromboplastin time (APTT), C-reactive protein (CRP), Fasting blood sugar (FBS), hemoglobin A1c (HbA1c), thyroglobulin (Tg), cholesterol, low-density lipoprotein (LDL), high-density lipoproteins (HDL), aspartate aminotransferase (AST), alanine aminotransferase (ALT), alkaline phosphatase (ALP), thyroid-stimulating hormone (TSH), blood urea nitrogen (BUN), creatinine, vitamin B12, and homocysteine (Hcy), Widal and Wright tests. Blood in EDTA tubes were centrifuged at 3000 g for 10 min at 4ºC to separate plasma. Collected samples were stored at -80°C until further analysis. Cytokine, Chemokine, TTR, Aβ Peptide, Hcy, and Vitamin B12 Assays ELISA kits for TTR, TNF-α, IL-1β, IL-1α, IL-2, IL-4, IL-6, IL-8, IL-12p70, IL-10, INF-γ, and a competitive ELISA kit for PGE2 were purchased from Abcam (Cambridge, UK). Commercial human MCP-1 and IP-10 ELISA kits were obtained from R&D Systems (Minneapolis, MN, USA). Hcy levels were enzymatically determined using the kit (Axis-Shield, Dundee, UK). Vitamin B12 was measured by an electrochemiluminescence (ECL) kit (Roche, Switzerland). Aβ 40 and Aβ 42 levels were determined using the Euroimmun ELISA kit (Lübeck, Germany). Duplicate assays were performed on collected plasma.

### Ethical Approval

Prior to enrollment, written informed consent was obtained from AD patients’ relatives. The present study was conducted under the approval of the Ethics Committee of SBMU (reference number: IR.SBMU.MSP. REC.1395.608.) and in accordance with ethical principles mentioned in the Declaration of Helsinki.

### Statistical Analysis

Qualitative and quantitative variables were expressed as percentage and mean±standard deviation (SD), respectively. Variables were examined for outliers and extreme values by box plot. Shapiro-Wilk test was employed to analyze normality. A partial correlation adjusted for age was performed. SPSS software version 24 (IBM Corp, Armonk, NY) was used for statistical analyses. P<0.05 was considered as statistically significant.

## Results

Among 93 subjects, females were 22 (56.4%), and males were 17 (43.6%). The mean age of patients was 69.95±5.43 years. Routine clinical markers including CBC, ESR, CRP, FBS, HbA1c, TG, Cholesterol, AST, ALT, ALP, Urea, Creatinine, PT (INR), APTT, and TSH were assessed to rule out the possibility of systemic or acute inflammation.Demographic data and routine biochemical test results are presented in (Table-1). Cognitive performance, including MMSE, CDR, ADAS-cog, and psychiatric symptoms NPI-Q scores are tabulated in (Table-2). Plasma Levels of Aβ 42, Aβ 40, Aβ 42/ 40 ratio, and TTR were 5.23±3.25, 69.28±25.63, 0.07±0.04, and 0.97±0.46, respectively, which are presented in (Table-3),(Table-4), and Figure-1. Plasma levels of TNF-a (12.94±8.16), INF-γ (24.56±15.77), IL-1a (3.95±2.26), IL-1b (11.26±6.33), IL-6 (19.05±10.86), and IL-10 (26.69±17.06) and other cytokines and chemokines such as MCP-1 (1141.87±468.9) were assessed, and data are summarized in (Table-3). The calculated ratios of TNF-a, IL-1b, and IL-6 to IL-10 are 0.83±0.95, 1.04±2.08, and 1.3±2.12, respectively (Table-4). Subsequently, obtained results were statistically analyzed to find a correlation between Aβ contents and its ratio, inflammatory molecules, pro- to anti-inflammatory cytokines, and cognitive test scores when adjusted for age. Those correlations were statistically significant in at least one parameter were tabulated (Table-5,Table-6,Table-7,Table-8). Based on our results, Aβ 40 was negatively correlated with IL-1b (r=-0.408, P=0.011) but positively correlated with NPI-severity and caregiver distress (r=0.399, P=0.026 and r=0.4, P=0.026, respectively). There were correlations between Aβ 42/40 ratio and both TTR (r=0.367, P=0.037) and INF-γ (r=-0.362, P=0.028). A negative correlation was observed between TTR and IP-10 (r=-0.322, P=0.049). Moreover, IL-1α was directly correlated with both Aβ 40 (r=0.379, P=0.019) and ADAS-cog (r=0.386, P=0.017) but was inversely correlated with IL-4 (r=-0.406, P=0.011). It could be suggested that elevated levels of IL-1α plays a role in AD pathogenesis or at least in disease exacerbation. Among chemokines, PGE2 was directly correlated with CDR (r=0.358, P=0.027), whereas inverse correlations were observed between MCP-1 and TNF-a/ IL-10 (r=-0.552, P=0.001), IL-1b/ IL-10 (r=-0.366, P=0.026), and IL-6/ IL-10 (r=-0.355, P=0.034) ratios. MMSE was negatively correlated with both PGE2 (r=-0.405, P=0.012) and TNF-α/IL-10 ratio (r=-0.35, P=0.037), while was not significantly correlated with TNF-a (r=-0.305, P=0.075) and IL-10 (r=0.113, P=0.512). Our results show a negative correlation between Hcy and vitamin B12 (r=-0.519, P=0.004).

## Discussion

Currently, there is no approved treatment for AD. Moreover, the molecular pathogenesis of AD has not been fully understood. Considering the possible role of inflammatory molecules in the early detection of AD, in this study, we focused on evaluating the correlation between plasma levels of pro- and anti-inflammatory cytokines, chemokines, TTR, and amyloid species with cognitive functions/neuropsychiatric symptoms to provide a substrate for finding a potential early-stage detection marker for AD. Neuroinflammation plays an important role in AD pathogenesis [26]. It is believed that in AD, microglia-mediated inflammation with the production of pro-inflammatory cytokines such as TNF-α, IL-1β, IL-6, and IL-12 occurs after Aβ deposition [26,27]. Bearing in mind that plasma levels of inflammatory molecules increase remarkably in AD, plasma levels of Aβ contents, cytokines, and chemokines in patients with moderate AD were measured to devise a comprehensive plasma-based sketch for target therapy. Disturbance of balance between Aβ production and clearance is known to be the leading cause of amyloid accumulation. Despite the substantial contribution of Aβ to AD, the underlying pathogenic mechanism has not yet been fully understood [28]. Interestingly, Aβ 40 was negatively correlated with IL-1b, but positively with IL-1a. Aβ 42/40 ratio, as a novel biomarker, mirrors the real-time clinical significance of amyloidogenesis, and various studies have demonstrated that this ratio declines significantly in AD patients [29,30]. Fandos et al.[31] found a negative correlation between the plasma ratio of Aβ 42/40 and brain Aβ level in cognitively normal cases. They also further proposed that this plasma ratio can serve as an early blood-based predi agnostic marker for AD [31]. TTR is a negative acute-phase protein; therefore, its serum level in AD patients is expected to decrease owing to persistent inflammation. It has been reported that CSF levels of TTR in AD patients fall drastically, and its concentration is negatively correlated with the AD stage [32,33]. Velayudhan et al . [34] achieved the same results for plasma levels of TTR in patients detected with AD compared with age-matched cognitively normal cases. They also conjectured that TTR plasma levels might predict of MMSE decline over a given period. They further proposed that the plasma level of TTR may serve as a suitable prognostic biomarker for AD [34]. However, our results showed no statistically significant correlation between TTR and MMSE. Moreover, a decline in TTR could disrupt the delicate balance of Aβ formation, and clearance towards deposition in CNS as TTR proteolytically cleaves Aβ and thereby decreases the formation of Aβ fibril. Our finding revealed a direct association between TTR and Aβ 42/40 ratio, which is in line with previous studies. Vitamin B12 deficiency is a common phenomenon rising with age [35]. It has also been suggested that there is a link between cognitive impairments and serum levels of vitamin Bs in the elderly [36]. Furthermore, it has been reported that elevated levels of Hcy are directly correlated with cognitive decline, brain atrophy, and dementia [37]. We observed a negative correlation between vitamin B12 and Hcy.

## Conclusion

AD is a chronic neurodegenerative disease that affects many people and has become one of the major health concerns worldwide. Obviously, successful treatments entail early diagnosis when individuals are asymptomatic. Current diagnostic biomarkers of AD (i.e.,CSF levels of Aβ and PET brain imaging) are believed to be present prior to the actual disease onset [38], but the former is invasive, and the latter is expensive. Therefore, finding novel, less invasive, more cost-effective, and efficient diagnostic biomarkers for AD is in desperate need. Furthermore, current medications have achieved limited success in alleviating symptoms of AD, which highlights the need for discovering alternative therapies [39]. Evidence from this study supports the potential of IL-1a as a plasma marker and provides a solid foundation for therapeutic strategies upon early AD detection. The limitations of our study were the sample size and, thereby, the lack of strong correlations.

## Acknowledgment

The authors gratefully acknowledge support from Shahid Beheshti University of Medical Sciences (SBMU). We sincerely appreciate Pars Teb pathobiology laboratory board, Dr. Ali Kharazian, Mohammadreza Bohluli Zanjani, and Mohsen Asadi. We are also extremely grateful to Dr. Ali Rahimipour, the head of the Clinical Biochemistry Department of SBMU.

## Conflict of Interest

None to declare.

**Table 1:** Demographic Data and Clinical Characteristics.

** Parameters**	** Values (Mean±SD)**
**Age (year)**	69.95±5.43
** Education (year)**	5±4.64
** Sex male (%)**	43.6%
**FBS (mg/dL)**	100.18±15.84
**HbA1c (%)**	4.98±0.68
**RBC (×1012/L)**	4.75±0.43
**Hgb (g/dL)**	13.82±1.42
**WBC (×109/L)**	6.13±1.51
**PLT (×109/L)**	218.51±62.92
** INR **	1
** APTT (s)**	30.18±1.93
**ESR (mm/h)**	11.23±6.43
**CRP (mg/L)**	2.59±1.74
**TG (mg/dL)**	124.72±64.1
** Cholesterol (mg/dL)**	183.15±42.39
** LDL (mg/dL)**	96.26±28.19
** HDL (mg/dL)**	49.08±9.87
** AST (U/L)**	21.36±8.19
**ALT (U/L)**	19.41±7.43
**ALP (U/L)**	184.23±45.81
**TSH (µIU/ml)**	2.28±1.42
**Urea (mg/dl)**	36±8.99
**Creatinine (mg/dl)**	1.03±0.25
**Vitamin B12 (pg/ml)**	465.27±493.4
**Hcy (µmol/L)**	25.81±21.17

**FBS:** Fasting Blood Sugar; **HbA1c:** Hemoglobin A1c; **RBC: **Red Blood Cell;** Hgb: **Hemoglobin; **WBC:** White Blood Cell; **PLT:** Platelet;** INR:** International Normalized ratio;** APTT: **Activated Partial Thromboplastin- Time;** ESR:** Erythrocyte Sedimentation Rate; **CRP:** C-Reactive Protein;** TG:** Triglycerides;** LDL:** Low-Desity Lipoprotein; **HDL:** High-Density Lipoproteins;** AST: **Aspartate Aminotransferase;** ALT:** Alanine Aminotransferase;** ALP:** Alkaline-Phosphatase;** TSH: **Thyroid Stimulating Hormone; **Hcy:** Homocysteine.

**Table 2:** Cognitive Test Scores.

** Clinical Tests**	** Score (Mean±SD)**
** MMSE**	15.72±4.14
** CDR**	7.17±2.5
**ADAS-cog**	52.79±13.87
**NPI- severity**	9.94±7.08
**NPI- caregiver distress**	12.09±10.68

**MMSE:** Mini-Mental State Examination;** CDR:** Clinical Dementia Rating;** ADAS-cog:** Alzheimer’s Disease Assessment Scale-cognitive subscale; **NPI- severity:** Neuropsychiatric Inventory- severity;** NPI- caregiver distress:** Neuropsychiatric Inventory- caregiver distress.

**Table 3:** Plasma Level of Amyloid Contents, TTR, Cytokines and Chemokines.

** Mediator (pg/ml)**	**Values (Mean±SD)**
** Aβ 42**	5.23±3.25
** Aβ 40**	69.28±25.63
** TTR**	0.97±0.46
** TNF-α**	12.94±8.16
** INF-γ**	24.56±15.77
** IL-1α**	3.95±2.26
** IL-1β**	11.26±6.33
**IL-2**	16.44±9.65
**IL-4**	7.19±4.38
**IL-6**	19.05±10.86
**IL-8**	18.57±11.38
** IL-10**	26.69±17.06
** IL-12p70**	27.29±13.26
** IP-10**	789.48±424.33
** MCP-1**	1141.87±468.9
** PGE2**	346.31±186.89

**Aβ:** Amyloid beta;** TTR:** Transthyretin;** TNF-α: **Tumor necrosis Factor α; **INF-γ: **Interferon-γ;** IL: **Interleukin; **IL-12p70: **Interleukin-12p70; **IP-10:** Interferon Gamma-Induced Protein 10;** MCP-1:** Monocyte Chemoattractant Protein-1;** PGE2: **Prostaglandin E2.

**Table 4:** Plasma Ratios of Ab 42 to 40 and Pro-inflammatory Cytokines (TNF-a, IL-1b and IL-6) to IL-10.

** Ratios**	**Values (Mean±SD)**
** Aβ 42/ 40**	0.07±0.04
** TNF-α/ IL-10**	0.83±0.95
** IL-1β/ IL-10**	1.04±2.08
** IL-6/ IL-10**	1.3±2.12

**Aβ:** Amyloid beta; **TNF-α:** Tumor Necrosis Factor α;** IL-1β: **Interleukin-1β; **IL-6: **Interleukin-6;** IL-10:**Interleukin-10.

**Table 5:** The Partial Correlation Adjusted for Age between Amyloid Contents and Other Parameters. Significance Level Was Considered as P<0.05.

**Aβ 42**	**Aβ 40**	**Aβ 42/40 ratio**
**r**	**P-value**	**r**	**P-value**	**r**	**P-value**
**Aβ 40**	0.473	0.003*	-	-	-0.022	0.899
**TTR**	0.141	0.42	-0.285	0.097	0.36	0.037*
**INF-γ**	-0.296	0.071	-0.115	0.492	-0.362	0.028*
**IL-1α**	-0.023	0.89	0.379	0.019*	0.047	0.874
**IL-1β**	-0.297	0.07	-0.408	0.011*	-0.144	0.394
**Tsh**	-0.452	0.006*	-0.348	0.038*	-0.017	0.92

**Aβ:** Amyloid beta;** TTR:** Transthyretin;** INF-γ: ** Interferon-γ;** IL-1α:** Interleukin-1α;** IL-1β:** Interleukin-1β;** TSH:** Thyroid Stimulating Hormone. *Significant Correlation.

**Table 6:** The Partial Correlation Adjusted for Age between Cognitive Tests and Other Parameters. Significance Level was Considered as P<0.05.

MMSE	CDR	ADAS-cog	NPI-severity	NPI-caregiver distress
r	P-value	r	P-value	r	P-value	r	P-value	r	P-value
Aβ 40	-0.185	0.266	0.122	0.476	0.167	0.317	0.399	0.026*	0.4	0.026*
TNF-α	-0.27	0.106	0.416	0.013*	0.236	0.161	-0.29	0.12	-0.338	0.068
IL-1α	-0.124	0.458	-0.076	0.651	0.386	0.017*	-0.095	0.61	-0.15	0.419
TNF-α/ IL-10 ratio	-0.35	0.037*	0.077	0.657	0.091	0.599	-0.082	0.672	-0.114	0.556
PGE2	-0.405	0.012*	0.358	0.027*	0.209	0.208	0.136	0.465	0.29	0.114
NPI-caregiver distress	-0.333	0.068	0.363	0.045*	0.449	0.019*	0.885	0.000*	-	-
TSH	0.223	0.185	-0.215	0.202	-0.307	0.065	-0.454	0.023*	-0.437	0.029*

**Aβ:** Amyloid-beta;** TNF-α:** Tumor Necrosis Factor α;** IL-1α: **Interleukin-1α;** IL-10:** Interleukin-10;** PGE2:** Prostaglandin E2;** TSH:** Thyroid Stimulating Hormone;** MMSE:** Mini-Mental State Examination; **CDR:** Clinical Dementia Rating; **ADAS-cog: **Alzheimer’s Disease Assessment Scale-cognitive subscale;** NPI- severity: **Neuropsychiatric Inventory-severity;** NPI- caregiver distress: **Neuropsychiatric Inventory- caregiver distress. *Significant Correlation

**Table 7:** The Partial Correlation Adjusted for Age between Plasma Pro-/Anti-inflammatory Ratios and Other Parameters. Significance Level Was Considered as P<0.05.

TNF-α/ IL-10 ratio	IL-1β/ IL-10 ratio	IL-6/ IL-10 ratio
r	P-value	r	P-value	r	P-value
TNF-α	0.173	0.334	-0.516	0.002*	-0.543	0.001*
IL-2	0.4	0.021*	0.289	0.082	0.126	0.464
IL-4	0.016	0.926	0.167	0.322	0.384	0.021*
IL-6	0.348	0.047*	0.022	0.901	0.381	0.022*
IL-8	0.098	0.569	0.021	0.9	0.403	0.015*
IL-1β/ IL-10 ratio	0.774	0.001*	-	-	0.896	0.000*
TNF-α / IL-10 ratio	-	-	0.774	0.001*	0.673	0.000*
MCP-1	-0.552	0.001*	-0.366	0.026*	-0.355	0.034*

TNF-α: Tumor Necrosis Factor α; IL-1α: Interleukin-1α; IL-1β: Interleukin-1β; IL-2: Interleukin-2; IL-4: Interleukin-4; IL-6: Interleukin-6; IL-8: Interleukin-8; IL-10: Interleukin-10; MCP-1: Monocyte Chemoattractant Protein-1; PGE2: ProstaGlandin E2. *Significant Correlation

**Table 8:** The Partial Correlation Adjusted for Age between Cytokines, Chemokines and TTR, Hcy and Vitamin B12. Significance Level Was Considered as P<0.05.

Correlation between	r	P-value
IL-1β and INF-γ	0.458	0.004*
IL-2 and MCP-1	-0.39	0.015*
IL-4 and IL-1 α	-0.406	0.011*
IL-6 and TSH	-0.336	0.045*
IL-8 and INF- γ	0.398	0.015*
IL-8 and IP-10	0.419	0.011*
TTR and IP-10	-0.322	0.049*
TTR and TSH	0.342	0.048*
Hcy and vit B12	-0.519	0.004*

TTR: Transthyretin; INF-γ: Interferon-γ; IL-1α: Interleukin-1α; IL-1β: Interleukin-1β; IL-2: Interleukin-2; IL-4:Interleukin-4; IL-6: Interleukin-6; IL-8: Interleukin-8; IL-10: Interleukin-10; IP-10: Interferon Gamma-induced protein 10; MCP-1:Monocyte Chemoattractant Protein-1; Hcy: Homocysteine; TSH: Thyroid Stimulating Hormone; Vit B12: Vitamin B12. *significant correlation
